# Soluble TNF-Like Weak Inducer of Apoptosis as a New Marker in Preeclampsia: A Pilot Clinical Study

**DOI:** 10.1155/2016/5930589

**Published:** 2016-02-17

**Authors:** Zeynep Kayaoglu Yildirim, Abdullah Sumnu, Neslihan Bademler, Elif Kilic, Gulay Sumnu, Serhat Karadag, Meltem Gursu, Aysegul Ozel, Gonca Batmaz, Seda Ates, Banu Dane, Savas Ozturk

**Affiliations:** ^1^Department of Obstetrics and Gynecology, Medical Faculty, Bezmialem Vakif University, Istanbul, Turkey; ^2^Department of Nephrology, Istanbul Medipol University, Istanbul, Turkey; ^3^Department of Biochemistry, Medical Faculty, Bezmialem Vakif University, Istanbul, Turkey; ^4^Department of Obstetrics and Gynecology, Haseki Training and Research Hospital, Turkey; ^5^Department of Nephrology, Haseki Training and Research Hospital, Istanbul, Turkey; ^6^Department of Nephrology, Medical Faculty, Bezmialem Vakif University, Istanbul, Turkey

## Abstract

*Introduction*. All findings of preeclampsia appear as the clinical consequences of diffuse endothelial dysfunction. Soluble tumor necrosis factor-like weak inducer of apoptosis (sTWEAK) was recently introduced as a TNF related cytokine in various inflammatory and noninflammatory disorders. sTWEAK was found to be related to endothelial dysfunction in patients with chronic kidney disease. In our study we aimed to compare sTWEAK levels in women with preeclampsia to corresponding levels in a healthy pregnant control group.* Materials and Methods*. The study was undertaken with 33 patients with preeclampsia and 33 normal pregnant women. The concentration of sTWEAK in serum was calculated with an enzyme linked immunosorbent assay (ELISA) kit.* Results*. Serum creatinine, uric acid, LDH levels, and uPCR were significantly higher in the patient group compared to the control group. sTWEAK levels were significantly lower in preeclamptic patients (332 ± 144 pg/mL) than in control subjects (412 ± 166 pg/mL) (*p* = 0.04).* Discussion*. Our study demonstrates that sTWEAK is decreased in patients with preeclampsia compared to healthy pregnant women. There is a need for further studies to identify the role of sTWEAK in the pathogenesis of preeclampsia and to determine whether it can be regarded as a predictor of the development of preeclampsia.

## 1. Introduction

Proangiogenic factors like placental growth factor (PGIF) and vascular endothelial growth factor (VEGF) must be in balance with antiangiogenic factors like soluble fms-like tyrosine kinase and soluble endoglin for a healthy placenta to grow. Increased production of antiangiogenic factors distorts this balance and causes endothelial dysfunction [1–4]. Endothelial dysfunction and organ damage caused by transfer to the maternal circulation of antiangiogenic factors due to placental ischemia resulting from abnormalities in the development of the placental vasculature are considered to be responsible for the pathophysiology of preeclampsia [5]. All findings of preeclampsia including hypertension, proteinuria, and vision defects appear as the clinical consequences of diffuse endothelial dysfunction [6, 7]. Proofs for endothelial dysfunction have been shown in many studies. Studies of increased fibronectin, factor-8 antigen and thrombomodulin, and decreased flow-mediated vasodilatation (FMD) are a few of these [8–10].

The soluble tumor necrosis factor-like weak inducer of apoptosis (sTWEAK, TNFSF12), a member of the TNF (tumor necrosis factor) superfamily, was recently introduced as a TNF related cytokine acting in various inflammatory and noninflammatory disorders [11]. It activates the nuclear factor kappa B pathway by binding to its receptor, Fn14, and mediates multiple effects, including cellular growth, proliferation, migration, differentiation, apoptosis, angiogenesis, fibrogenesis, and inflammation by stimulating expression of various proinflammatory cytokines and cellular adhesion molecules [12–15].

Jakubowski et al. demonstrated* in vivo* the regulatory role of sTWEAK in angiogenesis and its relation with VEGF [16]. They showed that the contact of endothelial cells with VEGF and fibroblast growth factor (FGF-2) induces the expression of Fn14 and that sTWEAK stimulates* in vitro* endothelial cell growth response acting in concert with VEGF and FGF-2 [17]. Besides its role in angiogenesis, sTWEAK was found to be related to endothelial dysfunction in patients with chronic kidney disease (CKD) and FMD and one of the independent predictors of endothelial damage [18].

To our knowledge, there is no data on the role of sTWEAK in preeclampsia, the pathogenesis of which is closely related to angiogenic mechanisms, and consequently endothelial dysfunction. We aimed in our study to compare sTWEAK levels in women with preeclampsia to corresponding levels in a healthy pregnant control group.

## 2. Materials and Methods

This case control study was performed between November 2013 and June 2014 after approval by the Bezmialem Vakif University Medical Faculty Ethics Committee.

### 2.1. Patients

The study was undertaken with 33 patients with preeclampsia and 33 normal pregnant women. Written informed consent was obtained from all participants. Preeclampsia was defined as the development of hypertension (systolic blood pressure (BP) ≥ 140 mmHg and/or diastolic BP ≥ 90 mmHg on two occasions at least 4 hours apart) and proteinuria (≥0.3 grams in a 24-hour specimen or urine protein/creatinine ratio (uPCR) ≥ 0.3) after the 20th week of gestation in a previously normotensive patient. Severe preeclampsia was defined as preeclampsia complicated by either a systolic BP ≥ 160 mm and/or a diastolic BP ≥ 110 mmHg (on 2 occasions at least 4 hours apart while the patient was on bed rest) and/or pulmonary edema and/or renal abnormality (progressive renal insufficiency; serum creatinine > 1.1 mg/dL) and/or cerebral/visual symptoms (persistent headaches, neurological symptoms, and visual disturbances) and/or hepatic abnormality (severe epigastric or right upper quadrant pain and/or liver transaminases at least twice the normal concentration) and/or platelet count < 100,000/microliter. According to the new preeclampsia criteria [19], patients with new onset hypertension (BP ≥ 140/90) without proteinuria were accepted to have severe preeclampsia if they had one of the above criteria. Exclusion criteria included twin or multiple pregnancies or any evidence of previous medical disease. 33 normal pregnant volunteers were randomly selected from patients who were admitted to the Obstetrics Department of the Medical Faculty of Bezmialem Vakif University at any time after the 20th week of gestation without any evidence of previous medical illness.

### 2.2. Laboratory Measurements

After overnight fasting, venous blood samples from patients were obtained to measure hemoglobin, thrombocyte, serum creatinine, uric acid, alanine transaminase (ALT), aspartate transaminase (AST), and lactate dehydrogenase (LDH) levels. These measurements, which are performed routinely in the follow-up of patients with preeclampsia, were done also for the control subjects. None of the preeclampsia patients and controls received any medications before blood sampling. Participants were asked to collect a random midstream urine sample for estimating the spot urinary protein/creatinine ratio (uPCR).

### 2.3. Measurement of sTWEAK Levels

Blood samples for sTWEAK measurements were collected in serum gel separator tubes and centrifuged for 10 minutes at a speed of 1500 g. Supernatant plasma was removed into polypropylene plastic tubes. Samples were taken and stored at −80°C until the time of analysis. The concentration of sTWEAK in serum was calculated with an enzyme linked immunosorbent assay (ELISA) kit, according to the protocols provided by the manufacturers (Human sTWEAK ELISA Kit, eBioscience Inc., San Diego, USA). A Multiskan FC® Microplate Photometer (Thermo Scientific, USA) was used for readings at 450 nm. The results were expressed as pg/mL.

### 2.4. Statistical Analysis

All variables were expressed as mean ± SD. *p* < 0.05 was considered to be statistically significant. Comparisons between two groups were assessed with Student's unpaired *t*-test and Mann-Whitney test or *χ* ^2^ test, as appropriate. Pearson or Spearman's rank correlation was used for univariate analysis, as appropriate. Multivariate linear regression analysis (backward method) was used to assess predictors of sTWEAK levels.

## 3. Results

The clinical data of the patient and the control groups are presented in Table 1. There was no statistically significant difference between the clinical characteristics of the two groups regarding age, weight, height, parity, gravidity, and abortus history (Table 1). The systolic blood pressure of the patient and the control groups was 156 ± 19 mmHg and 109 ± 14 mmHg, respectively (*p* < 0.001), while the corresponding levels of diastolic blood pressure were 97 ± 13 mmHg and 70 ± 11 mmHg (*p* < 0.001). Birth took place with caesarian operation in 89% of patients with preeclampsia, while this ratio was 64% in the control group (*p* = 0.02). Premature birth weight and low birth weight were more frequent in the patient group compared to the control group (*p* < 0.001).

The laboratory data of the groups are presented in Table 2. The laboratory analyses were performed at similar pregnancy weeks in both groups. The mean serum creatinine level and the mean uPCR in the patient group were 0.52 ± 0.10 mg/dL and 4597 ± 5466 mg/day, respectively. Serum creatinine, uric acid, LDH levels, and uPCR were significantly higher in the patient group compared to the control group. The sTWEAK level was significantly lower in preeclamptic patients (332 ± 144 pg/mL) than in control subjects (412 ± 166 pg/mL) (*p* = 0.04).

In the linear regression analysis model formed by the parameters potentially related to sTWEAK levels (age, the study group, the level of proteinuria, serum uric acid levels, and systolic blood pressure), only the study group was found to be an independent variable related to sTWEAK level (Table 3).

## 4. Discussion

The mean sTWEAK level was found to be lower in the preeclampsia group in this pilot study in which sTWEAK levels of preeclamptic patients were compared with control subjects (Table 2). The primary determinant of sTWEAK levels was found to be whether the individual was preeclamptic or not. Although there is no available data in the literature to compare with the results of the present study, in which sTWEAK was investigated in preeclampsia for the first time, it is possible to make indirect comments concerning data from different populations. sTWEAK is known to be active in the regulation of multiple biological functions, including induction of cell growth and angiogenesis, release of inflammatory cytokines, and stimulation of apoptosis [20]. Moreover, Yilmaz et al. found in their two studies a strong correlation between sTWEAK and endothelial dysfunction in patients with CKD [18, 21]. On the other hand, sTWEAK levels were found to be low not only in patients with CKD, but also in patient groups with high cardiovascular risk, including peripheral artery disease, abdominal aortic aneurysms, and ST elevation myocardial infarction [22–24]. Therefore, sTWEAK is being proposed as a cardiovascular risk factor [25]. The imbalance between angiogenesis and antiangiogenesis triggers endothelial dysfunction that is held to be responsible for all clinical findings in preeclampsia, which has been shown by many studies to increase cardiovascular risk [26]. In this aspect, the strong correlation found in our study between sTWEAK and preeclampsia supports the role of the endothelium in preeclampsia. There is need for far more studies to define the role of sTWEAK in quantitatively evaluating the degree of endothelial involvement.

There are not many studies on the relationship between pregnancy related diseases and sTWEAK in the literature. Recently Simón-Muela et al. showed decreased sTWEAK levels in patients with gestational diabetes compared to control subjects [27]. The authors related their finding to decreased insulin sensitivity, consistent with the previous report of Kralisch et al. [28].

It is not possible due to the observational nature of our study to say whether sTWEAK decreases as a result of endothelial dysfunction or whether it has a role in the pathogenesis of preeclampsia through steps in angiogenesis. In addition, the number of patients involved in our study was limited for comparing subgroups as early onset or late onset preeclampsia or preeclampsia with or without severe features. Another shortcoming of our study is the lack of data about other markers of endothelial dysfunction, such as flow-mediated dilation.

## 5. Conclusion

In conclusion, our study demonstrates that sTWEAK is decreased in patients with preeclampsia compared to healthy pregnant women. There is a need for further studies to clarify the role of sTWEAK in the pathogenesis of preeclampsia and to determine whether it can be regarded as a predictor of the development of preeclampsia.

## Figures and Tables

**Table 1:** Comparison of the groups regarding clinical characteristics.

	Patient (*n* = 33)		Control (*n* = 33)		*p* value
	Mean ± SD	Min–Max	Mean ± SD	Min–Max	
Age (years)	29.3 ± 4.9	18–39	28.4 ± 5.0	18–40	0.51
Weight (kg)	77.9 ± 15.1	60–130	75.4 ± 12.7	56–106	0.49
Height (cm)	159 ± 6	145–173	161 ± 5	151–169	0.38
Gravidity (*n*)	2.2 ± 1.4	1–8	2.2 ± 0.9	1–4	0.77
Parity (*n*)	0.9 ± 1.2	0–6	0.9 ± 0.7	0–2	0.71
Abortus (*n*)	0.2 ± 0.5	0–2	0.3 ± 0.5	0–2	0.30
Systolic BP (mmHg)	156 ± 19	128–200	109 ± 14	82–138	**<0.001**
Diastolic BP (mmHg)	97 ± 13	72–138	70 ± 11	46–94	**<0.001**
Gestational week at birth	34 ± 4	26–39	39 ± 1	38–94	**<0.001**
Birth weight (g)	2159 ± 964	470–3750	3375 ± 426	2570–4060	**<0.001**

**Table 2:** Comparison of the laboratory data of patients in both groups.

	Patient (*n* = 33)		Control (*n* = 33)		*p*
	Mean ± SD	Min–Max	Mean ± SD	Min–Max	
Gestational week	33 ± 4	26–39	32 ± 4	26–38	0.13
uPCR (mg/day)	4597 ± 5466	175–15578	180 ± 103	80–612	**<0.001**
Creatinine (mg/dL)	0.52 ± 0.1	0.4–0.8	0.4 ± 0.1	0.3–0.7	**<0.001**
Hemoglobin (g/dL)	11.2 ± 1.6	7.8–14.7	11.6 ± 1.2	8.9–13.8	0.27
Platelet (/*µ*L)	234 ± 77	92–404	232 ± 67	89–390	0.91
SGOT (U/L)	30 ± 31	8–188	19 ± 4	10–31	0.06
SGPT (U/L)	18 ± 19	3–98	13 ± 5	6–26	0.17
LDH (IU/L)	253 ± 134	150–846	186 ± 46	129–352	**0.01**
Uric acid (mg/dL)	4.9 ± 1.2	2.4–7.1	3.2 ± 1.0	1.9–5.9	**<0.001**
sTWEAK (pg/mL)	332 ± 144	89–686	412 ± 166	142–662	**0.04**

**Table 3:** Result of the linear regression model.

	*B*	Beta	*p*
Constant	−113.226		0.709
Group	211.296	0.663	**0.009**
Uric acid	13.591	0.124	0.472
Systolic BP	1.361	0.246	0.289
uPCR	0.011	0.312	0.061
Age	−3.242	−0.105	0.419
